# Statistics of Crime in France

**Published:** 1847-08

**Authors:** 


*Statistics of Crime in France.*—The Moniteur, government paper of France, states that during ten years, there were tried in the various criminal courts 41679 male prisoners, above the age of 25 years; among them were 33 priests, 33 lawyers, 75 notaries, 65 tip staffs, but not *a single medical practitioner.*

